# The complete mitochondrial genome of *Sinularia maxima* Verseveldt, 1971 (Octocorallia: Alcyonacea) using next-generation sequencing

**DOI:** 10.1080/23802359.2019.1674740

**Published:** 2019-10-07

**Authors:** You Chen, Ya-Ting Dan, Hao Lu, Pei-Zheng Wang, Alireza Asem, Weidong Li

**Affiliations:** a College of Ecology and Environment, Hainan Tropical Ocean University, Sanya, China;; b College of Fisheries and Life Sciences, Hainan Tropical Ocean University, Sanya, China;; c College of Ocean, Hainan University, Haikou, China

**Keywords:** Mitogenome, soft coral, *Sinularia maxima*, protein-coding genes, transfer RNA genes, ribosomal RNA genes

## Abstract

The mitochondrial genome of *Sinularia maxima* was completed using next-generation sequencing (NGS) method. The mitochondrial genome is a circular molecule of 18,730 bp in length. The gene arrangements include 14 protein-coding genes (PCGs), 2 ribosomal RNA genes, and 1 tRNA (*tRNA-Met*). The base composition is 30.18% A, 16.47% C, 19.35% G, and 33.99% T, with an A + T content of 64.18%. With regard to the phylogenetic analysis, members of genus *Sinularia* were clustered in different clades.

The genus *Sinularia* May, 1898 is one of the most widespread Octocorallia soft corals has been distributed in a wide range of habitats (Fabricius and Alderslade 2001). To date, the complete mitogenome of three species of *Sinularia* including *Sinularia ceramensis* (MK292119), *Sinularia* cf. *cruciate* (NC_034318) and *Sinularia peculiaris* (NC_018379) have been sequenced. In the present study, the complete mitochondrial genome of *Sinularia maxima* Verseveldt, 1971 (GenBank: MN485891) was analyzed using next-generation sequencing.

A specimen of *S. maxima* was collected from the South China Sea (West Island, Sanya, Hainan province, China; 18°14′ 8.75″N, 109° 22′39.10″ E) and stored in Hainan Tropical Ocean University Museum of Zoology (NO.0001-Sm). Taxonomical status of the specimen was identified by PuCAs-*mtMutS* (Benayahu et al. 2018) and PuCAs-28S (Quattrini et al. 2019). The whole DNA was extracted using Rapid Animal Genomic DNA Isolation Kit (Sangon Biotech Co., Ltd., Shanghai, CN; NO. B518221). A genomic library was made by paired-end (2 × 150 bp) next-generation sequencing, using the Illumina HiSeq X-ten sequencing platform (Asem et al. 2019). FastQC programme was utilized to check quality of sequencing reads (Andrews 2010) and the sequences were annotated and assembled to the *Sinularia* mitochondrial genome (*Sinularia ceramensis*, MK292119) with Spades v3.9.0 (Bankevich et al. 2012) and bowtie v2.2.9 (Langmead and Salzberg 2012). Putative tRNA gene was established using ARWEN (http://130.235.46.10/ARWEN/) online software. All genes were annotated based on gene order on the reference mitochondrial map using BLAST analysis (https://blast.ncbi.nlm.nih.gov). Additionally, to annotate PCGs and the position of start and stop codons were re-considered.

The complete mitogenome of *S. maxima* was 18,730 bp in length, with 14 protein-coding genes (PCGs), two ribosomal RNAs (rRNAs) and one transfer RNA (*tRNA-Met*). We found that *tRNA-Met* can be folded into typical clover-leaf secondary structures with 46.6% of GC content. The overall nucleotide composition of the major strand of the *S. maxima* mitogenome was as follows: 30.18% A, 16.47% C, 19.35% G, and 33.99% T, with a total A + T content of 64.18%.

The *tRNA-Met* and four protein-coding genes (*COX3*, *ATP6*, *ATP8,* and *COX2*) were located on the L strand. All PCGs began with common ATG start codon. Stop codons included eight TAG (*ND1*, *CYTB, ND6*, *ND3*, *ND2*, *ND5*, *COX3,* and *COX2*), five TAA (*ND4L*, *mutS*, *ND4*, *ATP6,* and *ATP8*) and a non-complete codons T- (*COX1*).

The *12S ribosomal RNA* and *16S ribosomal RNA* were encoded on H strand from 1583 to 2633 (1051 bp) and 9147 to 11115 (1969 bp), respectively, with *12S* having a rather higher G + C content (43.58% vs. 41.24%). A single overlap and the longest gap were found between and *ND2*/*ND5* (−13 bp) and *COX2*/*COX1* (112 bp), respectively.

A phylogenetic analysis of four completed *Sinularia* mitogenomes was established based on and an outgroup (*Leptogorgia capverdensis,* KY553145). The concatenated dataset for nucleotides contained 14 PCGs and two ribosomal RNAs. The maximum-likelihood (ML) phylogenetic analysis was performed using the software MEGA X (Kumar et al. 2018). Regarding phylogenetic tree, *Sinularia* is divided into two clads including *Sinularia* cf. *cruciate* + *Sinularia maxima* and *Sinularia peculiaris* + *Sinularia ceramensis* (Figure 1).

**Figure 1.**
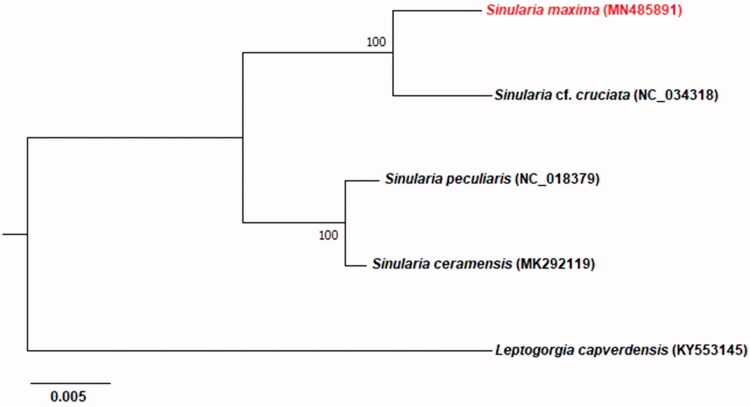
Phylogenetic tree showing the relationship among *S. maxima* and other members of order genus *Sinularia* based on maximum-likelihood (ML) approach. Numbers behind each node denote the bootstrap support values. The GenBank accession numbers are indicated on the right side of species names.
